# Dual Targeting of Smoothened, a Key Regulator in the Hedgehog Pathway, and BCR-ABL1 Effectively Eradicates Drug-Insensitive Stem/Progenitor Cells in Chronic Myeloid Leukemia

**DOI:** 10.3390/cells14191565

**Published:** 2025-10-09

**Authors:** Kelly A. Turner, Min Chen, Katharina Rothe, Donna L. Forrest, Xiaoyan Jiang

**Affiliations:** 1 Collings Stevens Chronic Leukemia Research Laboratory, Terry Fox Laboratory, British Columbia Cancer Research Institute, Vancouver, BC V6T 1Z3, Canada; kelly.turner@iwk.nshealth.ca (K.A.T.); mchen@bccrc.ca (M.C.); kat.rothe@utoronto.ca (K.R.); 2 Department of Medical Genetics, University of British Columbia, Vancouver, BC V6T 1Z3, Canada; 3 Leukemia/Bone Marrow Transplant Program of British Columbia, Vancouver, BC V6T 1Z3, Canada; dforrest@bccancer.bc.ca; 4 Department of Medicine, University of British Columbia, Vancouver, BC V6T 1Z3, Canada

**Keywords:** chronic myeloid leukemia, leukemic stem cells, tyrosine kinase inhibitors, therapy-resistance, Smoothened, Hedgehog pathway, Glasdegib (PF-04449913), Bostutinib

## Abstract

Overcoming drug resistance and targeting cancer stem cells remain challenges for curative cancer treatment. In particular, patients with chronic myeloid leukemia (CML) often require lifelong therapy with ABL1 tyrosine kinase inhibitors (TKIs), partly due to a persistent population of TKI-resistant leukemic stem cells (LSCs). Therefore, identifying specific pathways crucial for LSC maintenance is necessary. The Hedgehog (HH) pathway, especially the protein Smoothened (SMO), has been found to be essential for CML LSCs, but its role in TKI resistance is still largely unknown. We have now demonstrated that the expression of HH pathway genes *SMO* and *GLI2* is increased in CD34^+^ CML stem/progenitor cells compared to healthy counterparts, and is higher in TKI-nonresponders than in responders by transcriptome profiling and qRT-PCR analysis. Interestingly, they are most highly expressed in LSCs compared to progenitors and mature cells in TKI-nonresponders. Inhibition of SMO through genetic knockdown or with a potent, selective SMO inhibitor, Glasdegib, reduces the survival of cells from nonresponder patients. Notably, SMO inhibition also sensitizes TKI-nonresponder stem/progenitor cells to Bostutinib, a second-generation TKI, both in vitro and in a patient-derived xenotransplantation (PDX) model. These findings present a promising therapeutic target and a model for curative combination therapies in stem-cell-driven cancers.

## 1. Introduction

Chronic myeloid leukemia (CML) is a hematologic malignancy characterized by a reciprocal chromosomal translocation in a hematopoietic stem cell (HSC) [1,2]. The disease follows a triphasic course, beginning with the chronic phase (CP) and terminating in blast phase or blast crisis (BC) that closely resembles an aggressive acute leukemia and is refractory to treatment [2,3]. CML therapy was revolutionized with the development of the tyrosine kinase inhibitor (TKI) imatinib mesylate (IM), which explicitly inhibits ABL1 tyrosine kinase activity [4,5]. This leads to excellent outcomes for CML patients diagnosed in the CP [6,7]. However, IM and other advanced TKIs such as dasatinib or nilotinib are not curative, and not all patients respond to these drugs, particularly if treatment is initiated at later stages of the disease [7,8,9,10]. Primary and acquired resistance, and/or intolerance to therapy, also remain significant issues [11,12,13,14,15]. In addition, primitive, quiescent leukemic stem cells (LSCs) do not rely on BCR-ABL1 activity for their survival, and are often responsible for disease relapse once therapy with TKIs has been discontinued [15,16,17]. These studies highlight the necessity for therapies that can selectively target LSCs. One suggested approach has been the use of combination therapies—one drug to target the LSCs, and a TKI to target the mature leukemic cells, which constitute the bulk of the malignancy, such as to target TKI-resistant LSCs by inhibiting other pro-survival signaling pathways that are crucial to these cells, besides those controlled by BCR-ABL1 [7,10,15].

The Hedgehog (HH) pathway is critical for pattern formation in the developing embryo as well as adult homeostasis [18], and its role in human disease was first described when inherited inactivating mutations in *HH* were linked with holoprosencephaly (HPE), a midline defect involving the development of the forebrain and midface [19]. Interestingly, HH pathway activation has also been described as a driver in different forms of cancers [20,21,22,23,24]. The HH pathway in CML was not appreciated until two landmark papers demonstrated that intact HH signaling was required for CML LSC maintenance in mouse models. In both studies, it was observed that deletion or pharmacological inhibition of Smoothened (SMO) was detrimental to leukemia-initiating potential, maintenance, and self-renewal and LSC survival both in vitro and in vivo [13,14]. An attractive therapeutic strategy, therefore, would be the use of a TKI to target the bulk of the mature leukemic cells in combination with HH pathway suppression, through the inhibition of SMO, to eradicate the CML LSCs [24]. In particular, a specific SMO inhibitor, Glasdegib (GL, PF-04449913), has been developed and evaluated as an oral therapy for select hematologic malignancies and solid tumors [25,26,27,28], including acute myeloid leukemia (AML) and myelodysplastic syndromes (MDS) [25,26,27,28]. However, the extent to which the HH pathway is activated in CML patients is unknown, particularly between IM-responders and IM-nonresponders, and a better understanding of the reliance of CML LSCs on HH signaling is clearly needed.

Here, we demonstrated that HH pathway genes *SMO* and *GLI2* were upregulated in CML stem/progenitor cells from IM-nonresponders and were more sensitive to SMO inhibition compared with responders in vitro. The combination treatment of TKI Busotinib (BOS) and SMO inhibitor can significantly reduce engraftment of leukemic cells in mice, especially the engraftment of LSCs.

## 2. Materials and Methods

### 2.1. Patient Samples

Heparin-anticoagulated peripheral blood (PB) or bone marrow (BM) cells were collected from healthy donors and CML patients at diagnosis, before the initiation of TKI therapy, and retrospectively characterized as either IM-responders or IM-nonresponders according to the European LeukemiaNet CML treatment guidelines (Table S1) [3,29]. Mononuclear cells were isolated using Ficoll-Paque density gradient separation, and CD34^+^ cells were enriched using the EasySep™ Human CD34 Positive Selection Kit (STEMCELL Technologies, Vancouver, BC, Canada). Purity was verified by staining isolated cells with an anti-CD34 antibody conjugated to allophycocyanin (APC) (BD BioSciences, Mississauga, ON, Canada), followed by flow cytometric analysis, and cells were cultured as described previously [30].

### 2.2. Inhibitors

BOS and GL were obtained from Pfizer (New York, NY, USA) and reconstituted from powder in DMSO. Drugs were stored at −20 °C as 10 mM stock solutions. Working solutions were made by diluting the stock solution in 40% DMSO in Iscove’s MDM.

### 2.3. RNA Extraction and Quantitative Reverse Transcription PCR

Total RNA was extracted using TRIzol^®^ (ThermoFisher Scientific, Burlington, ON, Canada) according to the manufacturer’s instructions. An amount of 10 μg RNase-free glycogen was added as a carrier to assist with the visualization of the RNA pellet. Purified RNA was diluted in UltraPure™ DNase/RNase-free distilled water (ThermoFisher Scientific, Burlington, ON, Canada). In total, 100–200 ng RNA was reverse transcribed into cDNA using the Superscript^®^ VILO cDNA Synthesis Kit (ThermoFisher Scientific) according to the manufacturer’s instructions. The resulting cDNA was then diluted 1/10 in ddH_2_O. qRT-PCR was performed as previously described [30]. Primers used are listed in Table S2.

### 2.4. RNA Sequencing and Bioinformatic Analysis

Total RNA was isolated from CD34^+^ cells isolated by FACS from 3 normal BM (NBM) control samples, 3 IM-responder patient samples, and 3 IM-nonresponder patient samples from CP-CML patients as described [30]. Differential gene expression was quantified using *DESeq2* and other bioinformatics analyses as described [30,31]. To minimize a high false discovery rate, *p*-values were adjusted using the Benjamini-Hochberg (B-H) method [32]. The differential expression of HH pathway-associated genes was investigated using Bioconductor *DESeq2* analysis. Significance of differentially expressed genes was determined by Benjamini-Hochberg-adjusted *p*-value < 0.05 (Table S3).

### 2.5. Viability and Apoptosis Assays

Cell viability was assessed after 72 h using the trypan blue exclusion method. Total viable cell numbers were determined using a Neubauer hemocytometer and expressed as a percentage relative to untreated controls. Apoptosis analysis was performed using the Annexin V Apoptosis Detection Kit APC (eBioscience, San Diego, CA, USA) according to the manufacturer’s instructions. Cells were harvested after 72 h exposure to inhibitors and stained with an Annexin V-APC antibody and PI. Flow cytometry analysis was performed, and the proportion of apoptotic cells was then determined [15].

### 2.6. Colony-Forming Cell (CFC) and Re-Plating Assay

CD34^+^ cells inhibitors were plated in duplicate 35 mm cultures dishes (STEMCELL Technologies) per 1 mL of MethoCult™ H4230 (STEMCELL Technologies) medium with growth factors (20 ng/mL IL-3, 20 ng/mL IL-6, 50 ng/mL G-CSF, 50 ng/mL GM-CSF, and 2 U/mL EPO, all from STEMCELL Technologies). After a 12–14-day incubation at 37 °C and 5% CO_2_, erythroid, granulopoietic, and mixed colonies were enumerated, and the numbers of test arms were expressed as a percent of the numbers measured in parallel untreated control assays [15,33].

For re-plating, the resulting colonies from the CFC were isolated, individual cells counted, and 10^4^ cells per 1 mL of MethoCult™ H4230 with growth factors and without drugs were re-plated in duplicate. Colonies were scored 7 days later in the same manner as above. The CFCs derived from the re-plating experiment were expressed as a percent of the total number of cells harvested at the end of the 2-week re-plating experiment, back-calculated per the original input of 10^3^ cells per 1 mL MethoCult™ into the initial CFC, and normalized to an untreated control.

### 2.7. Long-Term Culture-Initiating Cell (LTC-IC) Assay

Detailed procedure has been previously described [33]. 2 × 10^4^ CD34^+^ cells were cultured for 6 weeks in MyeloCult™ H5100 medium (STEMCELL Technologies) on human mouse stromal cells. Inhibitors were added at the outset of the experiment, and one-half medium changes were performed once per week without the addition of inhibitors. After 6 weeks, the cells were harvested and 10^4^ viable cells per 1 mL Methocult™ H4230 with growth factors were plated in CFC assays as described [15,33].

### 2.8. Lentiviral-Mediated SMO shRNA Knockdown in CD34^+^ CML Patient Samples

Lentiviral constructs pLKO.1 containing SMO shRNAs (scrambled shRNA or shRNA specific to SMO, either TRN0000014365 (SMO#1) or TRCN0000014367 (SMO#2)) were purchased from Sigma. Lentiviral particles were packaged in 293T cells as described [34]. CD34^+^ CML primary cells were pre-cultured for 16 h in the presence of 4 growth factors, as described above, and then infected with lentiviral particles containing scrambled shRNA or shRNA specific to SMO for 6 h. The infected cells were then selected by puromycin (5 μg/mL) for three days before they were harvested for biological assays.

### 2.9. Xenotransplantation Experiments Using PDX Models

2.5 × 10^6^ highly purified CD34^+^ IM-nonresponder patient cells per mouse were intravenously injected into irradiated female NRG mice (800cGy). Six weeks later, transplanted mice were orally treated with 100 mg/kg BOS alone, 100 mg/kg GL alone, a combination of these two inhibitors, or vehicle solutions (control) for 3 weeks. Leukemic cell burden was measured via BM aspiration at 10 and 16 weeks after transplantation from each mouse. Immunophenotypes of engrafted cells were determined with fluorescently labeled CD45 (FITC and PE, eBioscience), CD34 (AF647, BioLegend, San Diego, CA, USA), CD38 (BV711, BioLegend), CD33 (PE-Cy7, eBioscience), CD15 (PE-Cy7, eBioscience), and CD19 (APC-Cy7, eBioscience). Animal experiments were performed in the Animal Resource Centre of the BC Cancer Research Centre using procedures approved by the Animal Care Committee of the University of British Columbia (Vancouver).

### 2.10. Statistical Analysis

Results are presented as the mean ± standard error of the mean from at least three independent experiments. Group differences were assessed using the 2-tailed Student *t*-test or one-way ANOVA using the Bonferroni correction for multiple comparisons.

## 3. Results

### 3.1. HH-Associated Genes, SMO and GLI2, Are Highly Elevated in CD34^+^ CML Cells, Particularly in IM-Nonresponders

**Figure 1:**
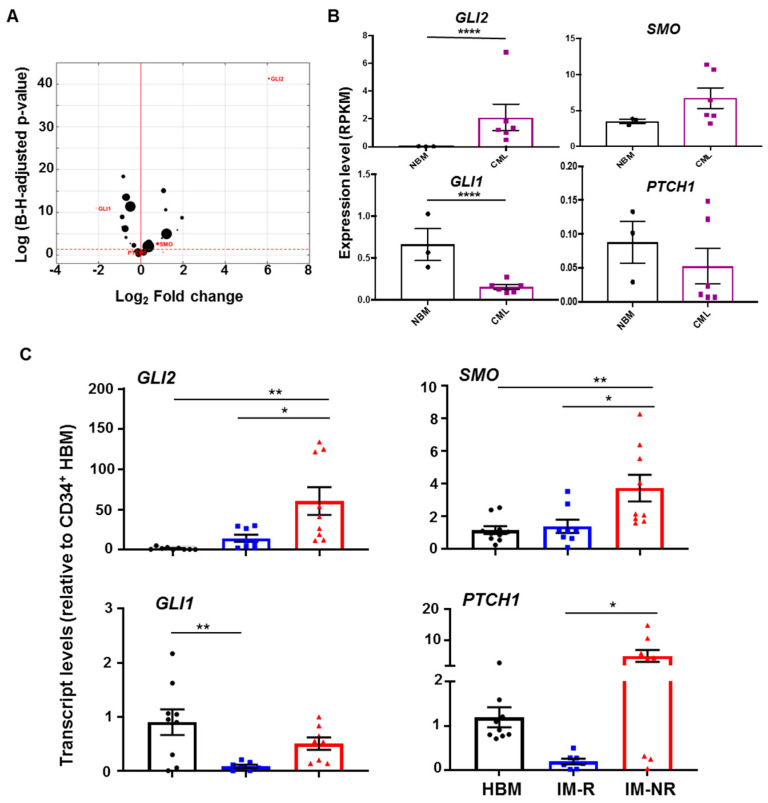
**Identification of differentially expressed HH pathway-associated genes in CD34^+^ CML cells.** (**A**) Volcano plot displaying differential gene expression of 42 HH pathway-associated genes between CD34^+^ CML patient samples (n = 6) and normal bone marrow (NBM) controls (n = 3) by Bioconductor DESeq2 analysis. Highly differentiated genes are highlighted in red. The size of the dots reflects the mean expression level in absolute read counts and is normalized for sequencing depth. The broken red line indicates the significance threshold (Benjamini-Hochberg-adjusted *p* < 0.05). (**B**) Expression levels of the main HH pathway genes between NBM and CML, measured in RPKM. (**C**) qRT-PCR validation on CD34^+^ cells from 8 NBM, 8 IM-responders, and 9 IM-nonresponders. Black box: NBM, blue box: IM-responders (IM-R), and rad box: IM-nonresponders (IM-NR). * = *p* < 0.05; ** = *p* < 0.01; **** = *p* < 0.0001.

To determine whether the HH pathway is differentially expressed in CD34^+^ CML patient cells compared to CD34^+^ cells from healthy individuals, we examined transcript levels of 42 HH pathway-associated genes based on our RNA sequencing (RNA-seq) analysis from three CD34^+^ normal bone marrow (NBM) controls and six CD34^+^ CML patient samples obtained at diagnosis [30,35,36]. Using Bioconductor *DESeq2* analysis, 17 genes were significantly differentially expressed (>2-fold, *p* < 0.05) in CD34^+^ cells from the CML samples compared with CD34^+^ NBM controls (Figure 1A, Table S3). In particular, transcripts for one of the principal HH pathway genes, *GLI2*, were particularly elevated in the CD34^+^ CML cells (>10-fold, *p* < 0.0001, Figure 1A,B). These results suggest that the HH pathway is more active in CD34^+^ CML cells compared with CD34^+^ NBM cells.

Next, gene expression differences between IM-responder and IM-nonresponder patient samples were compared. Of the six CD34^+^ CML patient samples used for the RNA-seq analysis, three were retrospectively characterized as IM-responders, and three were IM-nonresponders. Transcripts of *GLI2* and *SMO*, two of the positive regulators of the HH pathway, were more elevated in IM-nonresponders compared with IM-responders (up to 8-fold, Figure S1). qRT-PCR validation of the RNA-seq results confirmed this pattern of expression of HH pathway-associated genes, with increased expression of *GLI2*, *SMO*, and *PTCH1* between IM-responders (n = 8) and IM-nonresponders (n = 9, *p* < 0.05, Figure 1C, Table S1). Taken together, these results suggest that the HH pathway is involved in regulating the properties of stem/progenitor cells from IM-nonresponder patients compared with IM-responder patients.

**Figure 2:**
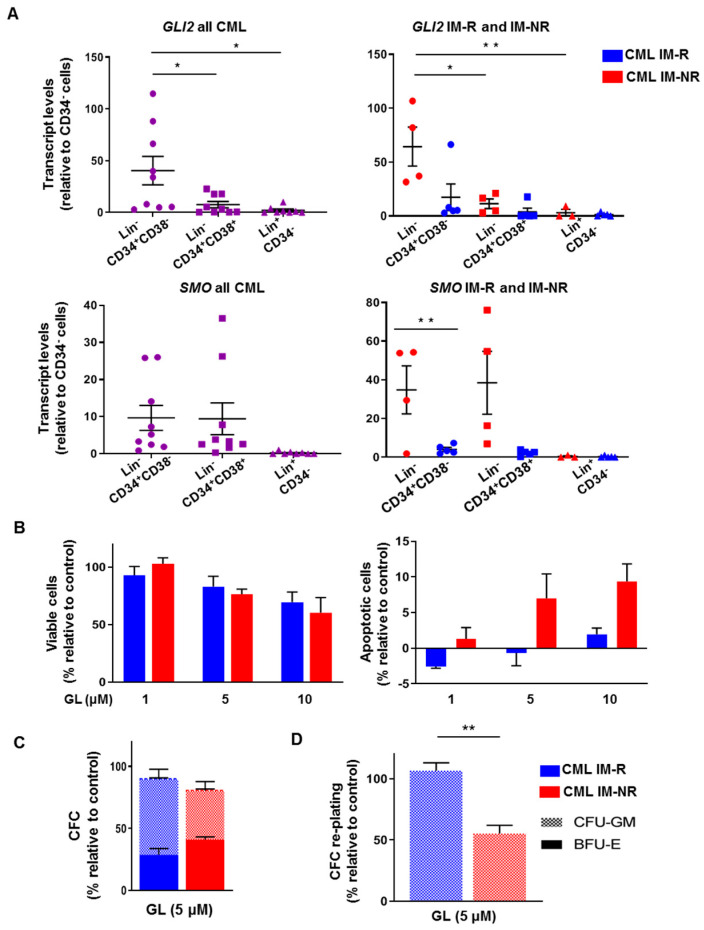
**Transcript levels of *GLI2* and *SMO* are increased in LSCs in IM-nonresponders, correlating with their response to SMO inhibition.** (**A**) Relative expression of *GLI2* and *SMO* measured by qRT-PCR in the Lin^-^CD34^+^CD38^-^ stem-enriched subpopulation (circles), Lin^-^CD34^+^CD38^+^ progenitor subpopulation (squares), and Lin^+^CD34^-^ mature hematopoietic subpopulations (triangles) across all CML patient samples (n = 9, left). Expression levels in IM-nonresponders (n = 5, red symbols) and IM-responders (n = 4, blue symbols, right). Each dot represents a single patient sample. (**B**) Viability assay of CD34^+^ cells after 72 h of exposure to increasing doses of GL, using the trypan blue exclusion method (n = 3). Apoptosis assay in CD34^+^ cells after 72 h of treatment with increasing GL doses, using PI and Annexin V staining followed by FACS analysis (n = 3). (**C**) CFC assay performed on CD34^+^ CML cells, plated in methylcellulose with growth factors and inhibitors, with colonies counted and typed after 14 days (n = 3). (**D**) CFC re-plating assay using a portion of colonies from the initial CFC, re-plated into fresh methylcellulose with growth factors but no drugs, with colonies counted and typed after 7–10 days (n = 3). Colony types are indicated by a pattern based on morphology. BFU-E = burst-forming unit-erythroid; CFU-GM = colony-forming unit-granulocyte/macrophage. * = *p* < 0.05; ** = *p* < 0.01.

Interestingly, relative to CD34^-^ mature cells, *GLI2* transcripts were highly increased in the Lin^-^CD34^+^38^-^ (stem-enriched) fraction (38-fold), compared with the Lin^-^CD34^+^38^+^ (progenitor) subpopulation (7-fold), and the difference between the stem-enriched fraction and the progenitors and mature cells was statistically significant (*p* < 0.05, Figure 2A). In particular, this effect was amplified when comparing the differences in *GLI2* expression in the different CD34 subpopulations between IM-responders and IM-nonresponders (>4-fold, *p* < 0.05, Figure 2A). *SMO* transcripts were also present at higher levels in both the stem-enriched fraction and the progenitor cells relative to CD34^-^ CML cells (>10-fold) and this effect was exacerbated when comparing IM-nonresponders and IM-responders, particularly in the stem-enriched and the progenitor fractions of nonresponders (7-fold, *p* < 0.05, Figure 2A).

These results demonstrate that both *SMO* and *GLI2* are most highly expressed in the stem-enriched subpopulation in both IM-responder and nonresponder patient samples, compared with more mature cell subsets. Most interestingly, they were more highly expressed in the stem-enriched fraction from IM-nonresponder patient samples compared with IM-responders, suggesting that IM-nonresponders utilize the HH pathway as a pro-survival mechanism to evade eradication by TKIs.

### 3.2. A Selective SMO Inhibitor, Glasdegib, Is More Effective at Targeting CD34^+^ CML Cells from IM-Nonresponders In Vitro

Since CD34^+^ cells from IM-nonresponders highly expressed *SMO* and *GLI2* compared with IM-responders, and represent a more clinically interesting patient population, the sensitivity of these cells in response to the SMO inhibitor Glasdegib (GL, PF-04449913), a potent and orally bioavailable inhibitor, was determined [26,27,28]. Interestingly, we observed that CD34^+^ cells from IM-nonresponders showed a slight decrease in cell viability, but more apoptosis compared with IM-responders by GL treatment in short-term assays (Figure 2B and Figure S2). To examine the effects of SMO inhibition on primitive leukemic cells on a relatively long term, the colony-forming cell (CFC) assay was utilized. Compared with CD34^+^ CML cells from IM-responder patients, IM-nonresponders were slightly more sensitive to GL (Figure 2C). The drug treatment did not appear to differentially target particular colony types in either IM-responder or IM-nonresponder samples. To investigate the effects of GL on more primitive leukemic cells, re-plating assays were performed. A significant reduction in CFC yields in the re-plated cells was observed with IM-nonresponder samples compared to IM-responder cells (2-fold, *p* < 0.01, Figure 2D).

Overall, these results suggest that more primitive leukemic cells are more sensitive to SMO inhibition, and IM-nonresponders are more sensitive to SMO inhibition compared with IM-responders. These results are in concordance with the gene expression data observed and support the hypothesis that the cell subsets that express key HH pathway genes to a greater extent are more sensitive to HH pathway inhibition.

### 3.3. Dual Inhibition of SMO and BCR-ABL1 Effectively Inhibits Proliferation and Long-Term Clonogenic Activities of CML Stem/Progenitor Cells In Vitro

**Figure 3:**
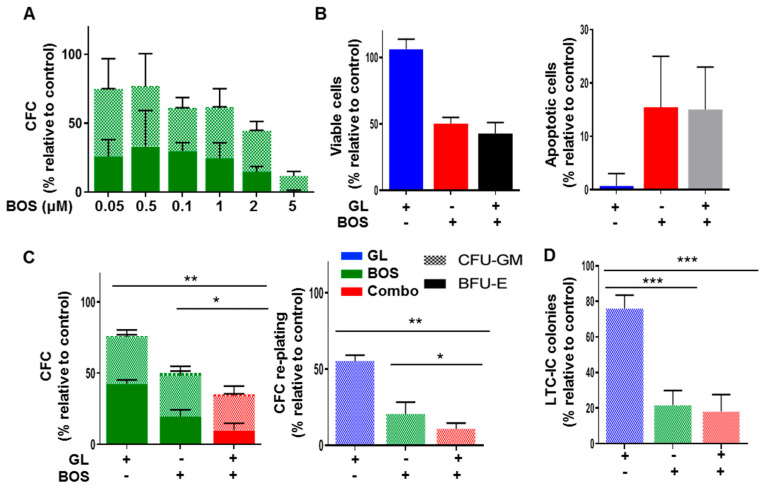
**Dual inhibition of SMO and BCR-ABL1 suppresses the growth of CD34^+^ IM-nonresponder cells in vitro.** (**A**) Viability assay after 72 h using the trypan blue exclusion method. Cells were exposed to GL ± BOS (n = 3). (**B**) Apoptosis assay after 72 h using Annexin V/PI staining followed by FACS analysis at the same doses used for the viability experiment (n = 3). (**C**) CFC assay using GL or BOS or a combination (n = 3), followed by a re-plating experiment. (**D**) LTC-IC-derived CFC measurements were performed on CD34^+^ CML cells by exposing cells to GL or BOS or both for one week, then conducting half medium changes without drugs for 6 weeks. The cells were subsequently harvested, and a portion was plated into CFC, with colonies counted after 2 weeks. Bar patterns indicate colony types based on morphology. BFU-E = burst-forming unit-erythroid, CFU-GM = colony-forming unit-granulocyte/macrophage. * = *p* < 0.05; ** = *p* < 0.01; *** = *p* < 0.001.

To determine whether combining SMO inhibition with a more effective second-generation TKI, Bosutinib (BOS), is more effective at targeting IM-nonresponder stem/progenitor cells, several in vitro assays were performed on CD34^+^ CML cells from IM-nonresponders. The IC50 dose of BOS was determined to be 2.5 µM using CFC assays on CD34^+^ cells from six IM-nonresponders (Figure 3A). In short-term viability and apoptosis assays, the drug combination was not better than single agents in CD34^+^ cells from three IM-nonresponder samples (Figure 3B and Figure S3). However, a significant decrease in colony number was observed with the combination treatment, compared to BOS alone, with colonies almost completely abolished upon re-plating by the combination (<10% colonies remaining), compared to GL and BOS alone (*p* < 0.01, Figure 3C). In the long-term culture-initiating cell (LTC-IC)-derived CFC assay (an in vitro stem cell assay), BOS alone or in combination reduced CFC output by 82%, and this difference was significant (*p* < 0.01, Figure 3D). Notably, both drugs alone and together showed no toxicity (BOS up to 5 µM and GL up to 20 µM) in CD34^+^ NBM cells, suggesting a therapeutic window for targeting CML cells while sparing normal hematopoietic cells (Figure S4).

### 3.4. Glasdegib Treatment Results in the Decreased Expression of GLI2 Selectively in Stem Cell-Enriched CML Cells

**Figure 4:**
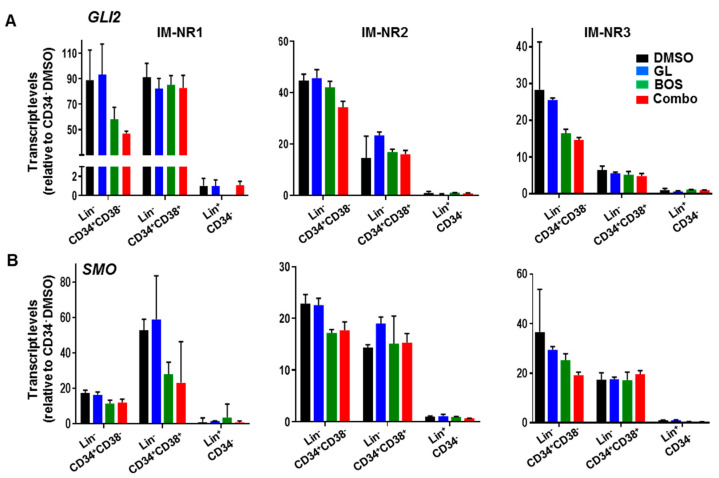
**Dual inhibition of SMO and BCR-ABL1 reduces GLI2 expression specifically in LSCs** (**A**,**B**). This is demonstrated by qRT-PCR analysis of GLI2 and SMO expression in CD34^+^ subpopulations from three IM-nonresponder patient samples treated with GL or BOS, alone or combined, for 16 h in serum-free medium with or without inhibitors.

To demonstrate that GL could specifically repress the HH pathway via SMO inhibition, qRT-PCR was performed to track gene expression changes in HH pathway members SMO and GLI2 after treatment. Based on previous results indicating that the HH pathway, and particularly GLI2, was most highly expressed in the stem-enriched (Lin^-^CD34^+^CD38^-^) subpopulation, it was hypothesized that HH pathway inhibition, and consequently GLI2 suppression, would be most potent in this subpopulation relative to the others. Indeed, after overnight exposure to GL in combination with BOS, a decrease in expression of GLI2 was observed in the Lin^-^CD34^+^CD38^-^ population with the combination treatment in all three IM-nonresponder samples (up to 35%, Figure 4A). However, no decreases were detected in any of the treatments of the progenitor (Lin^-^CD34^+^CD38^+^) and mature (CD34^-^) subpopulations in the same samples. Changes in SMO expression were not evident in the CD34-subpopulations from these patient samples, as expected, since SMO itself is not a downstream target of the GLI transcription factors (Figure 4B). These findings suggested that the combination treatment was most effective at reducing the expression of GLI2, a critical regulator downstream of SMO, selectively in the stem-enriched subpopulation.

### 3.5. Knockdown of SMO in CD34^+^ CML Stem/Progenitor Cells Sensitizes Their Responses to BOS

**Figure 5:**
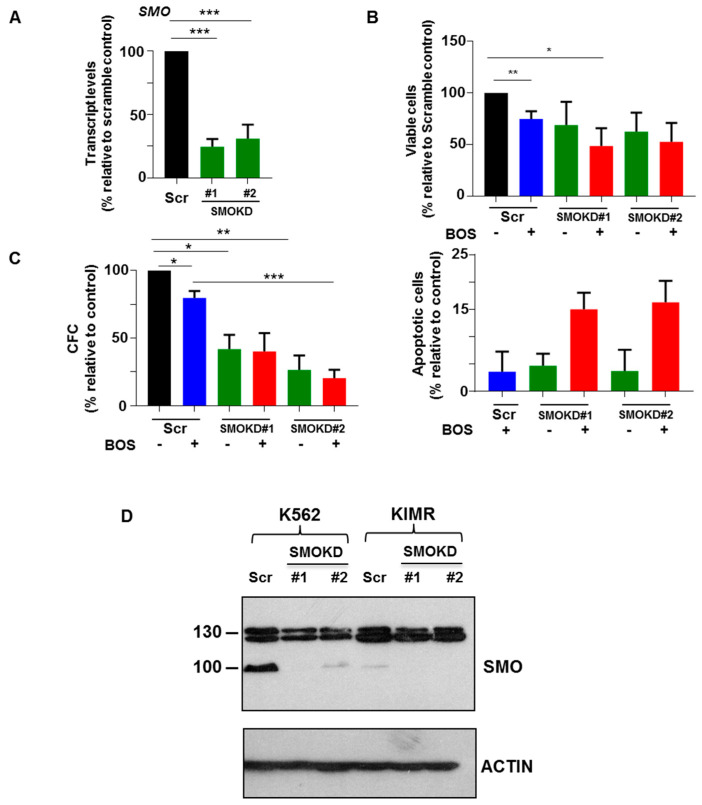
**Knockdown of SMO in CD34^+^ stem/progenitor cells reduces cell viability and CFC output.** (**A**) SMO transcript levels in lentiviral-specific SMO shRNA-transduced CD34^+^ IM-nonresponder cells compared to scramble-transduced cells, measured by qRT-PCR analysis. (**B**) Viability and apoptosis assays in the same SMO knockdown cells, with or without BOS treatment. (**C**) CFC assay in these SMO knockdown cells, with or without BOS. (**D**) SMO protein expression levels in lentiviral-specific SMO shRNA-transduced K562 and K562 IM-resistant (KIMR) cells compared to scramble-transduced cells, measured by Western blot analysis. * = *p* < 0.05; ** = *p* < 0.01; *** = *p* < 0.001.

Combination treatment of BOS and GL has demonstrated more potent inhibitory killing effects on CD34^+^ CML stem/progenitor cells, especially towards IM-nonresponders. To further confirm that the combination effects in these CD34^+^ CML cells were due to the specific inhibition of SMO activity, we stably knocked down SMO using a lentiviral-mediated system. Two constructs carrying short hairpin RNA (shRNA) specifically targeting SMO and a scrambled control were cloned into a lentiviral pLKO.1 vector, and these two clones showed successful knockdown of SMO (up 75%) in CD34^+^ IM-nonresponder cells by qRT-PCR analysis (*p* < 0.05, Figure 5A). Their reduced protein expression was confirmed in K562 and K562-resistant cells (Figure 5D). Knockdown of SMO in CD34^+^ cells decreased cell viability compared to scramble control cells. The inhibitory effects were more potent when these cells were treated with BOS (*p* < 0.05, Figure 5B). More apoptotic cells were detected in SMO knockdown cells treated with BOS, compared to untreated SMO knockdown cells or scramble control cells treated with BOS (Figure 5B and Figure S5). In the CFC assay, a significant reduction in colonies was also observed in SMO knockdown cells compared to control cells in both shRNAs-targeted cells (*p*< 0.05, Figure 5C). Taken together, the inhibitory effects of SMO knockdown in CD34^+^ IM-nonresponder cells indicated that SMO-related signal pathways have an essential role in regulating TKI-insensitive stem/progenitor cell survival.

### 3.6. Dual Inhibition of SMO and BCR-ABL1 in CD34^+^ Stem/Progenitor Cells Resulted in Reduced Engraftment of Leukemic Cells in a Patient-Derived Xenotransplantation (PDX) Model

**Figure 6:**
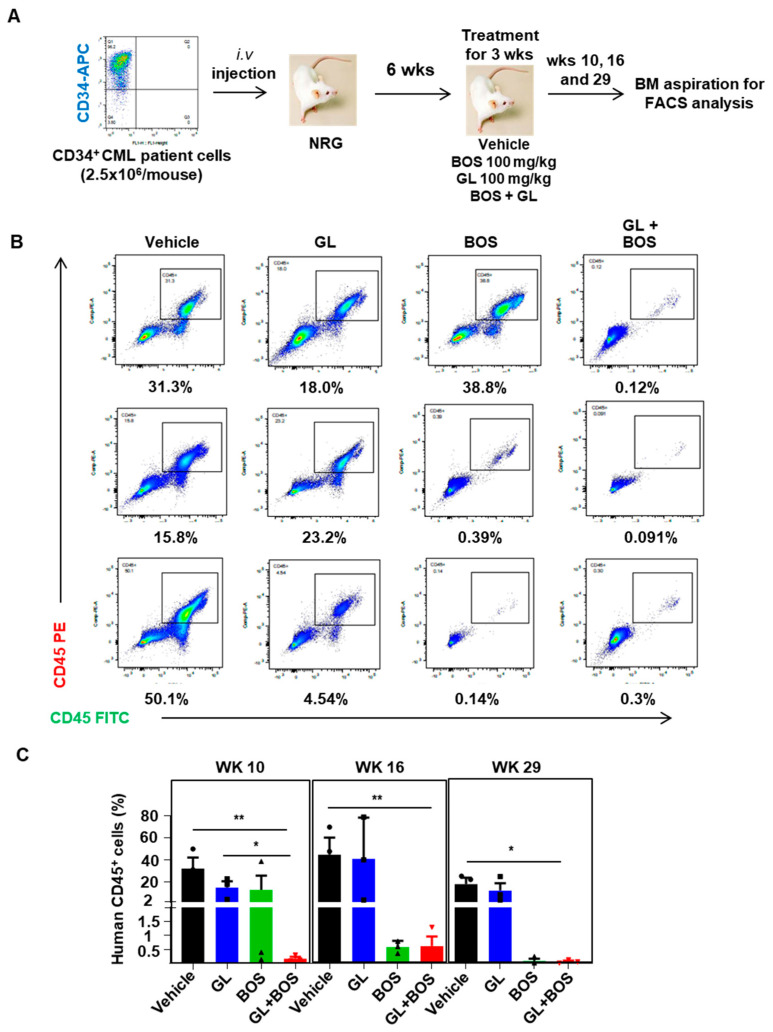
**Combination treatment of BOS and GL reduces engraftment of human CD45^+^ leukemic cells in mice**. (**A**) Schematic of experimental design using a PDX model. 2.5 × 10^6^ CD34^+^ IM-nonresponder cells were intravenously injected into each lethally cesium-irradiated NRG mouse, treated with inhibitors, and their engrafted leukemic cells were analyzed. (**B**) FACS profiles showed engrafted human CD45^+^ cells in mice BM from each treatment group after one week of oral gavage treatment (3 mice per group). (**C**) The percentage of human CD45^+^ cells in BM aspiration from mice of each treatment group was determined at week 10, week 16, and week 29 post-transplantation by FACS analysis. * = *p* < 0.05; ** = *p* < 0.01.

**Figure 7:**
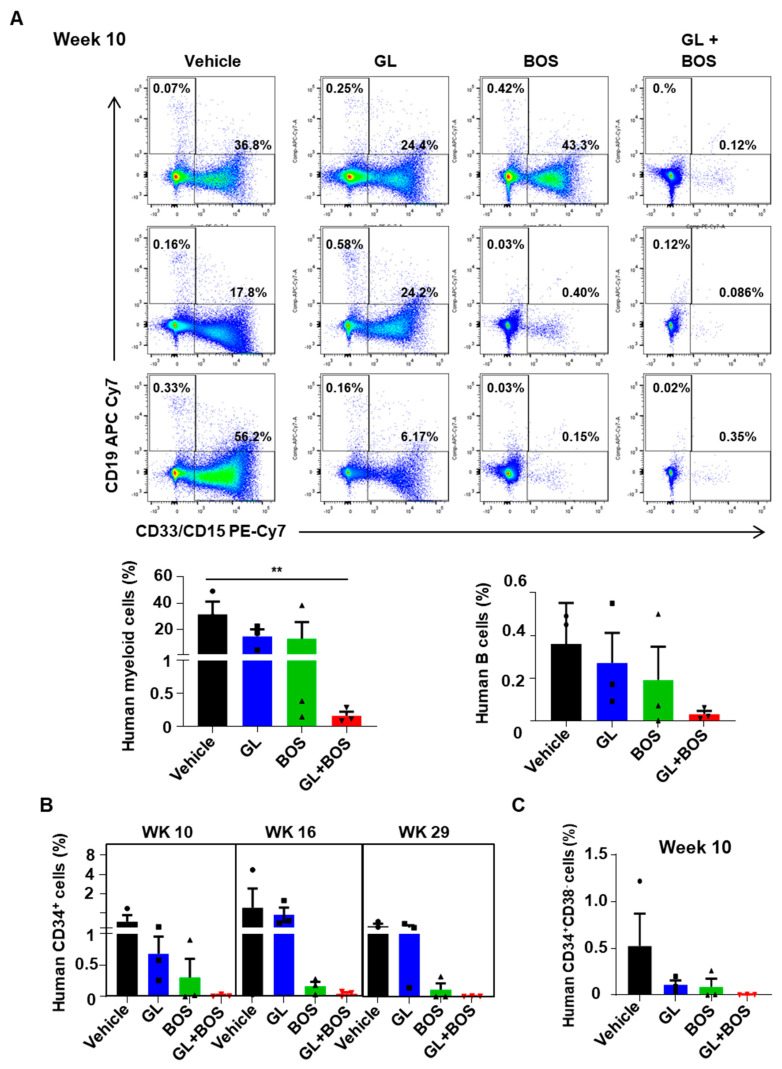
**Combination treatment of BOS and GL eliminates long-term LSCs in vivo.** (**A**) FACS analysis showing engrafted CD33^+^CD15^+^ and CD19^+^ cells in each mouse across different treatment groups at 10 weeks post-transplantation (left panel). The percentage of human CD33^+^CD15^+^ myeloid cells and CD19^+^ B cells in BM aspiration is shown in the right panel. (**B**) The percentage of human CD34^+^ cells in BM from mice in each treatment group at weeks 10, 16, and 29 after transplantation. (**C**) The percentage of Lin^-^CD34^+^CD38^-^ cells in BM aspiration at week 10 post-transplantation. ** = *p* < 0.01.

To evaluate the effectiveness of combination treatment in eliminating leukemic cells, especially leukemic stem/progenitor cells in vivo, CD34^+^ IM-nonresponder cells were intravenously injected into lethally irradiated NRG mice, followed by oral administration of inhibitors for three weeks (Figure 6A). Analysis of the bone marrow (BM) engraftment at 10 weeks post-transplantation (one week after completing the oral treatment) showed a significant decrease in human CD45^+^ cells in mice treated with the BOS and GL combination compared to those treated with either BOS or GL alone (0.17% vs. 13% for BOS or 22% for GL, Figure 6B,C). Most of the CD45^+^ cells in the BM at 10 weeks were CD33^+^CD15^+^ myeloid cells (Figure 7A). Additionally, the number of engrafted CD34^+^ stem/progenitor cells and the more primitive Lin^-^CD34^+^CD38^-^ LSCs declined in mice treated with either BOS or GL alone, with fewer of these cells in mice receiving both BOS and GL (Figure 7B,C). Notably, the long-term engraftment capacity of human leukemic cells at 29 weeks post-transplantation was significantly reduced in the combination-treated mice, with very low levels of human CD45^+^ and CD34^+^ cells compared to mice treated with vehicle or GL alone (Figure 6C and Figure 7B). Mice treated with BOS displayed reduced human CD45^+^ leukemic cells in BM at 16 and 29 weeks post-transplantation (Figure 6C). Although there was a minor difference between mice treated with BOS alone and those with the combination after 16 and 29 weeks, BOS plus GL treatment demonstrated more potent killing effects in eliminating human leukemic cells at earlier time points (Figure 6C). These findings indicate that the second-generation TKI BOS is highly effective at targeting CML stem/progenitor cells, and combining GL and BOS further enhances the elimination of IM-nonresponder cells in a PDX model.

## 4. Discussion

ABL1 TKIs have revolutionized CML treatment for managing early-stage CML; however, innate and acquired TKI resistance mechanisms remain a significant challenge in achieving a cure for the disease. This includes the persistence of drug-insensitive LSCs that rapidly generate therapy-resistant clones and initiate disease relapse in CML patients [15,16,17,37,38]. Particularly, the HH pathway, which is critical during embryonic development, is essential for CML initiation and LSC maintenance [13,14,23]. This has prompted interest in further studies of the HH pathway as a therapeutic target in CML. In addition, it was unknown whether the principal HH pathway genes might be differentially expressed in CML cells of IM-responders and IM-nonresponders. In this study, we first examined transcript levels of the HH pathway-associated genes based on our RNA-seq analysis from CD34^+^ CML patient cells obtained at diagnosis compared to CD34^+^ healthy control cells [30]. *SMO* and *GLI2* transcript levels, among 11 differentially expressed genes found in the HH pathway, were highly increased in IM-nonresponders compared with IM-responders. A validation in additional CML patients further supported this result. Interestingly, these two genes were differentially expressed between primitive and mature subpopulations, where they were more highly expressed in the stem-enriched subpopulation compared to both progenitors and the more mature subpopulations. These results suggested that the HH pathway, particularly SMO and GLI2, may be critical in regulating the properties of LSCs from IM-nonresponder patients compared with IM-responders. Additional studies to increase sample size, including patient samples in the later stages of disease (e.g., AP and BC samples), will provide more insight into this significant finding. These findings were supported by a study demonstrating that overexpression of the HH pathway and cell cycle regulatory genes correlates with leukemic progression. In particular, *GLI2* played a role in regulating CML LSC dormancy [39]. It was found that CD34^+^ progenitor cells transduced with *GLI2* preferentially reside in the G0 phase of the cell cycle, compared with cells transduced with the vector control. In addition, a study using IM-resistant patient samples found that autocrine HH signaling promotes drug resistance by upregulating BCL-2 [40]. This study demonstrated that these resistant cells had upregulated BCL-2 levels and that inhibiting either SMO or BCL-2 could resensitize leukemic cells to IM. It has also been reported that the activation of SMO significantly influences metabolic pathways by promoting LSC survival, proliferation, and resistance to drug treatment in AML and CML, since HH signaling is aberrantly activated, leading to increased cell growth and survival, often by promoting LSC quiescence and differentiation into more vulnerable states, or by interacting with other pathways like PI3K/AKT/mTOR [41,42,43]. Thus, the HH-mediated regulation of LSC properties, including their dormancy state and metabolic vulnerability, may be contributing factors to TKI resistance to therapy.

We then tested our hypothesis that IM-nonresponder LSCs would be more sensitive to HH pathway suppression via the SMO inhibition, since the HH pathway is aberrantly expressed in CML cells and may constitute a mechanism by which the LSCs are maintained. Interestingly, when CD34^+^ cells from IM-responders and IM-nonresponders were treated with the SMO inhibitor GL, IM-nonresponders were indeed more sensitive to SMO inhibition compared with IM-responders, concerning cell survivability, apoptosis, and colony-forming potential. Most interestingly, a dual treatment strategy comprising the second-generation TKI BOS in combination with GL in CD34^+^ IM-nonresponder cells showed significant improvements in reducing colony-forming ability and re-plating potential compared with either agent alone. Notably, BOS alone was quite effective in targeting stem/progenitor cells from IM-nonresponders in vitro, which were not observed by using IM or the other second generation of TKIs [44,45,46,47]. These results were further supported by the genetic knockdown of SMO in these cells. These results indicated that more primitive CML cells are more severely impacted by SMO inhibition, particularly in IM-nonresponders, and a combination of GL and BOS is effective to eradicate these cells.

To demonstrate that GL can specifically suppress the HH pathway through SMO inhibition, we used qRT-PCR to monitor gene expression changes in downstream HH pathway members after treatment. Our observations showed that the HH pathway, particularly *GLI2*, was most highly expressed in the stem-enriched subpopulation. We hypothesized that inhibiting the HH pathway, and thus suppressing GLI2, would be most effective in this subpopulation compared to others. Indeed, *GLI2* transcripts were greatly reduced in the stem-enriched population following GL or combination treatment. This reduction was not observed in the progenitor or mature subpopulations. This suggests that the combination treatment was most effective at reducing *GLI2* expression—a key regulator downstream of SMO—specifically in the stem-enriched cells. We did not observe significant changes in SMO transcripts within the CD34 subpopulations after treatment, as expected, since SMO itself is not a downstream target of GLI transcription factors. Although all patient samples showed the highest *GLI2* expression in the stem-enriched subpopulation, the levels varied between patients. Indeed, while using patient samples for this study provided significant direct evidence of how the HH pathway was affected in IM-nonresponder patient cells, the relatively small sample size and limited cell numbers for additional molecular studies are limitations. It would also be interesting to determine molecular and biological changes in GLI2 downstream target genes involved in the effect of SMO inhibition on the HH pathway. Overall, the in vitro and molecular findings indicate that HH activity is essential for maintaining LSCs, and that dual inhibition of BCR-ABL1 and the HH pathway, especially targeting SMO, may offer an effective strategy to target drug-resistant LSCs.

Cancer stem cells are known to be resistant to chemotherapy and targeted therapies, but the mechanisms of action remain largely unknown [48,49,50]. In this study, we provide direct evidence that the HH pathway is involved in both the survival and the long-term propagating activity of CML stem cells. Strikingly, the highest expression of *GLI2* and *SMO* was found in CML stem cells, correlating with phenotypical consequences of SMO and ABL1 inhibition being most profound in the long-term co-culture system with stromal cells and in the PDX model, which measures functional LSC activities both in vitro and in vivo. The combination approach was not toxic to healthy, adult BM counterparts, indicating that cancer-specific pathways are being targeted. From a clinical and translational perspective, the clinical effectiveness of a combination therapy comprising a TKI and SMO inhibitor as an LSC-targeted therapy in CML remains to be determined. In a phase 1 study of GL in CML patients, one patient with BC-CML achieved a partial cytogenetic response (pCyR). Still, there were no other good responders, suggesting that efficacy-focused studies with combination therapies are needed [51]. Two other clinical trials on dual TKI and SMO inhibitor treatments have also been conducted for CML, with one assessing the combination of dasatinib with BMS-933923 and the other evaluating nilotinib with LDE225. Both trials reported moderate responses for these combination therapies, but also reported adverse side effects in patients [23,52,53]. Interestingly, we have now demonstrated that a combination of GL with a potent TKI, BOS, is effective in targeting IM-resistant cells as well as LSCs. Thus, a combination of GL with BOS would be a promising approach for a clinical trial.

## 5. Conclusions

Our study is the first, to our knowledge, to demonstrate that principal HH pathway genes, and most notably *GLI2* and *SMO*, are differentially expressed between IM-responders and IM-nonresponders, particularly LSCs. Furthermore, we show that subsequent HH pathway suppression is more detrimental to IM-nonresponders cells compared with responders, both in vitro and in vivo. These results support the hypothesis that the HH pathway is more critical for LSCs from IM-nonresponders, and may represent a mechanism by which drug-resistant cells evade eradication by TKIs.

## Data Availability

Data are contained within the article and Supplementary Materials.

## Supplementary Materials

The following supporting information can be downloaded at: https://www.mdpi.com/article/10.3390/cells14191565/s1, Table S1. Characteristics of CML patient samples used in this study. Table S2. Specific primer sequences for qRT-PCR. Table S3. Differential expression of 42 HH pathway-associated genes between CD34^+^ NBM and CD34^+^ CML samples by RNA-seq analysis. Figure S1. Several key HH pathway genes are differentially expressed in CD34^+^ cells from IM-nonresponders compared with IM-responders. A) Expression of the principal HH pathway genes between normal BM (HBC), IM-responders (IM-R), and IM-nonresponders (IM-NR) from RNA-seq. Figure S2. SMO inhibitor treatment increased the number of apoptotic cells in CD34^+^ IM nonresponder cells. Representative FACS profiles in CD34^+^ cells after 72 hours of treatment with increasing GL doses, using PI and Annexin V staining, followed by FACS analysis from an IM-nonresponder patient. Figure S3. Dual inhibition of SMO and BCR-ABL1 increased apoptosis in CD34^+^ IM nonresponder cells. Representative FACS profiles in CD34^+^ cells after 72 hours of treatment with GL or BOS, alone or in combination, using PI and Annexin V staining, followed by FACS analysis from an IM-nonresponder patient. Figure S4. GL and BOS are well-tolerated by CD34^+^NBM cells. CFC assay in 3 normal bone marrow (NBM) samples tested with various doses of GL ± BOS. The patterns within the bars correspond to the proportion of colony types based on morphology. BFU-E = burst-forming unit-erythroid, CFU-GM = colony-forming unit-granulocyte/macrophage. Figure S5. Knockdown of SMO with BOS treatment increased apoptosis in CD34^+^ IM nonresponder cells. Representative FACS profiles in CD34^+^ cells knocked down with two different constructs, with or without treatment of BOS for 72 hours, using PI and Annexin V staining, followed by FACS analysis from an IM-nonresponder patient. scramble control = Scr.
