# Supplementary material for: Dual Targeting of Smoothened, a Key Regulator in the Hedgehog Pathway, and BCR-ABL1 Effectively Eradicates Drug-Insensitive Stem/Progenitor Cells in Chronic Myeloid Leukemia
Source: Cells. 2025 Oct 9;14(19):1565. doi: 10.3390/cells14191565 (PMC12523657; doi:10.3390/cells14191565)
Supplement: Supplementary file 1 [file cells-14-01565-s001.zip › cells-3849992-supplementary.pdf]

Table S1. Characteristics of CML patient samples used in this study.

Patient number	Sex	Age at Diagnosis	WBC at Diagnosis (10^3/mL)	Disease Stage at Diagnosis	IM Response
CML 1	M	45	392.3	CP-CML	NR
CML 2	M	22	212.8	CP-CML	NR
CML 3	M	N/A	N/A	CP-CML	NR
CML 4	F	66	492.0	CP-CML	NR
CML 5	M	55	676.6	CP-CML	NR
CML 6	M	48	140.0	CP-CML	NR
CML 7	M	21	202.9	CP-CML	NR (BC)
CML 8	M	24	88.7	CP-CML	NR
CML 9	F	19	355.5	CP-CML	NR
CML 10	M	28	225.5	CP-CML	NR
CML 11	M	27	480	CP-CML	NR
CML 12	F	N/A	197	CP-CML	NR
CML 13	M	42	244.2	CP-CML	NR
CML 14	F	56	301.6	CP-CML	R
CML 15	M	23	324.4	CP-CML	R
CML 16	F	78	192.3	CP-CML	R
CML 17	M	N/A	N/A	CP-CML	R
CML 18	M	32	353.2	CP-CML	R
CML 19	M	57	450	CP-CML	R
CML 20	M	54	77	CP-CML	R
CML 21	M	59	37.6	CP-CML	R

Table S2. Specific primer sequences for qRT-PCR

Primer	Sequence (5′→3′)
β2M-F	TAGCTGTGCTCGCGCTACT
β2M-R	TCTCTGCTGGATGACGTGAG
PTCH1-F	CCACAGAAGCGCTCCTACA
PTCH1-R	CTGTAATTTCGCCCCTTCC
SMO-F	TTACCTTCAGCTGCCACTTCTACG
SMO-R	GCCTTGGCAATCATCTTGCTCTTC
GLI1-F	TTCCTACCAGAGTCCCAAGT
GLI1-R	CCCTATGTGAAGCCCTATTT
GLI2-F	GGATTCCAGCTGTCTTGTCTT
GLI2-R	CCAGAGAGGATGCCCATAAAC

Table S3. Differential expression of 42 HH pathway-associated genes between CD34^{+} NBM and CD34^{+} CML samples by RNA-seq analysis

Gene Name	Sequencing Mean Expression (absolute normalized read count)	Fold Change (log_2)	Benjamini-Hochberg Adjusted p-value
GLI2	630	6.1	1.11E-18
KIF3B	2500	-0.83	1.05E-08
ARRB1	4100	1.1	2.80E-07
CUL3	9000	-0.69	1.30E-06
NUMB	6300	-0.75	1.35E-06
CSNK1A1	17000	-0.50	1.19E-05
GLI1	76	-2.1	1.73E-05
STK36	1900	1.2	2.57E-05
FBXW11	3500	-0.88	0.00013
EVC	2200	2.0	0.00016
BOC	5.4	-3.8	0.00138
SUFU	1700	-0.84	0.00150
GSK3B	7700	-0.75	0.00212
CCND1	510	1.7	0.00293
MYC	18000	1.2	0.00694
KIF7	1500	-0.71	0.01638
EVC2	560	1.0	0.01992
GLI4	630	0.35	0.04043
PRKACA	2800	0.32	0.05779
LRP2	66	1.1	0.05804
BMI1	8400	0.38	0.06114
PTCH2	370	-0.50	0.06701
SMO	1500	0.79	0.07276
ARRB2	3500	-0.32	0.10527
PRKACB	22000	0.35	0.13193
IHH	5.1	-1.4	0.15359
KIF3A	970	0.27	0.25332
BCL2	3400	0.35	0.28759
CDO1	8.2	-1.2	0.29298
KIF3C	2000	-0.25	0.43424
CUL1	7600	-0.13	0.46816
GLI3	120	1.0	0.51055
PTCH1	38	-0.72	0.53682
DHH	6.2	-0.79	0.54207
HIP1	6800	0.14	0.56271
SPOPL	6700	-0.11	0.74558

Gene Name	Sequencing Mean Expression (absolute normalized read count)	Fold Change (log_2)	Benjamini-Hochberg Adjusted p-value
GPR161	340	-0.17	0.75306
GAS1	36	0.36	0.79083
SHH	2.8	0.029	0.98689
PRKACG	0.19	0.12	NA
HHIP	230	-6.9	NA
IGF2	77	-7.6	NA

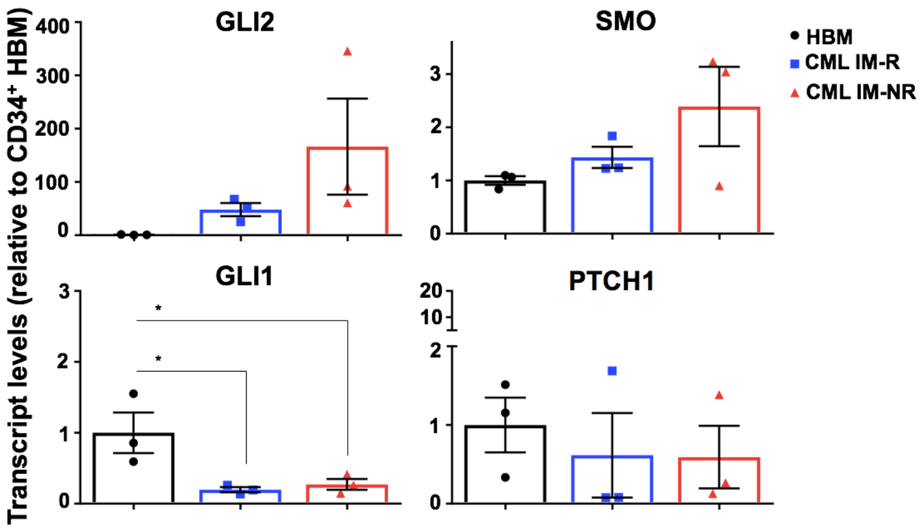

Figure S1. Several key HH pathway genes are differentially expressed in CD34^{+} cells from IM-nonresponders compared with IM-responders. A) Expression of the principal HH pathway genes between normal BM (HBC), IM-responders (IM-R), and IM-nonresponders (IM-NR) from RNA-seq.

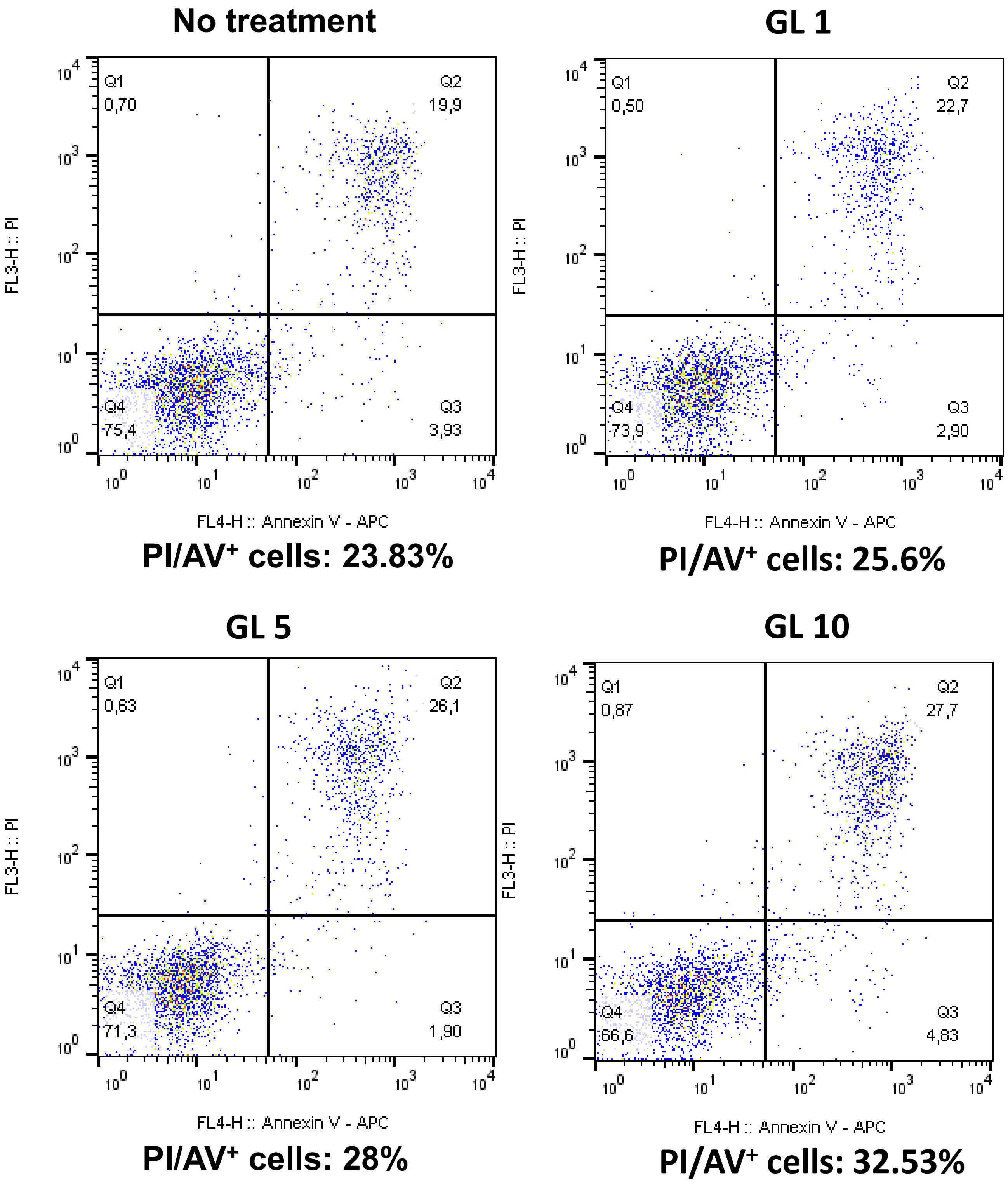

Figure S2. SMO inhibitor treatment increased the number of apoptotic cells in CD34+ IM-nonresponder cells. Representative FACS profiles in CD34+ cells after 72 hours of treatment with increasing GL doses, using PI and Annexin V staining, followed by FACS analysis from an IM-nonresponder patient.

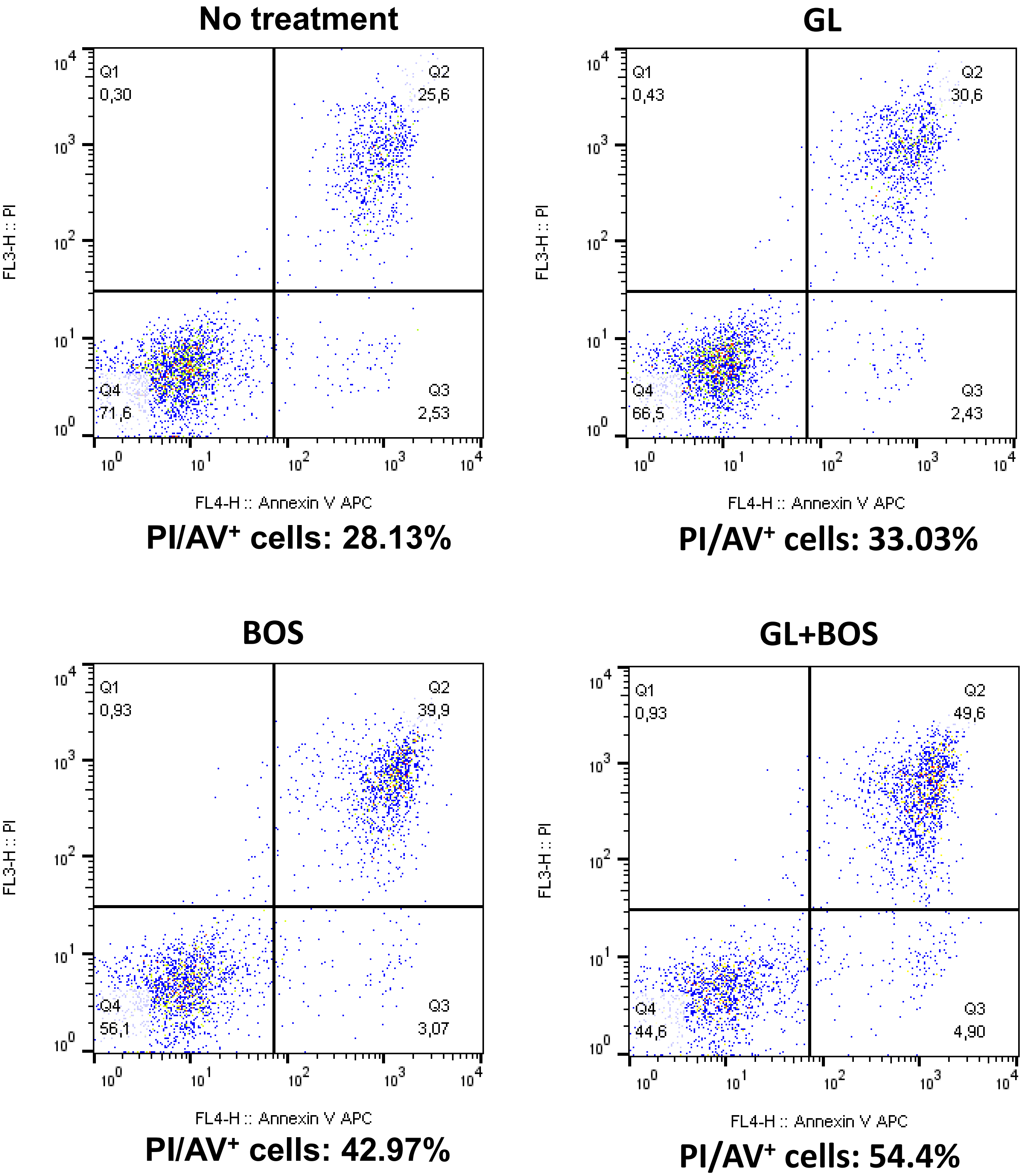

Figure S3. Dual inhibition of SMO and BCR-ABL1 increased apoptosis in CD34^{+} IM-nonresponder cells. Representative FACS profiles in CD34^{+} cells after 72 hours of treatment with GL or BOS, alone or in combination, using PI and Annexin V staining, followed by FACS analysis from an IM-nonresponder patient.

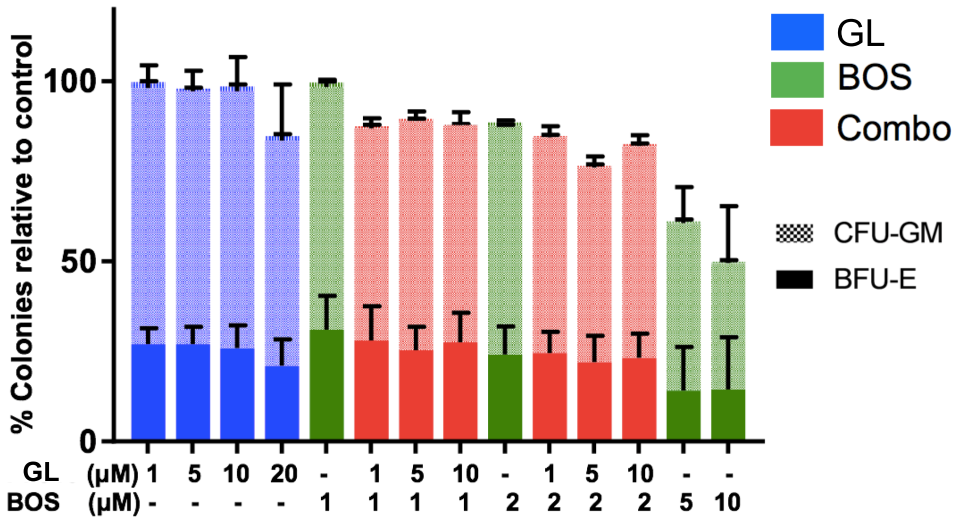

Figure S4. GL and BOS are well-tolerated by CD34+ NBM cells. CFC assay in 3 normal bone marrow (NBM) samples tested with various doses of GL ± BOS. The patterns within the bars correspond to the proportion of colony types based on morphology. BFU-E = burst-forming unit-erythroid, CFU-GM = colony-forming unit-granulocyte/macrophage.

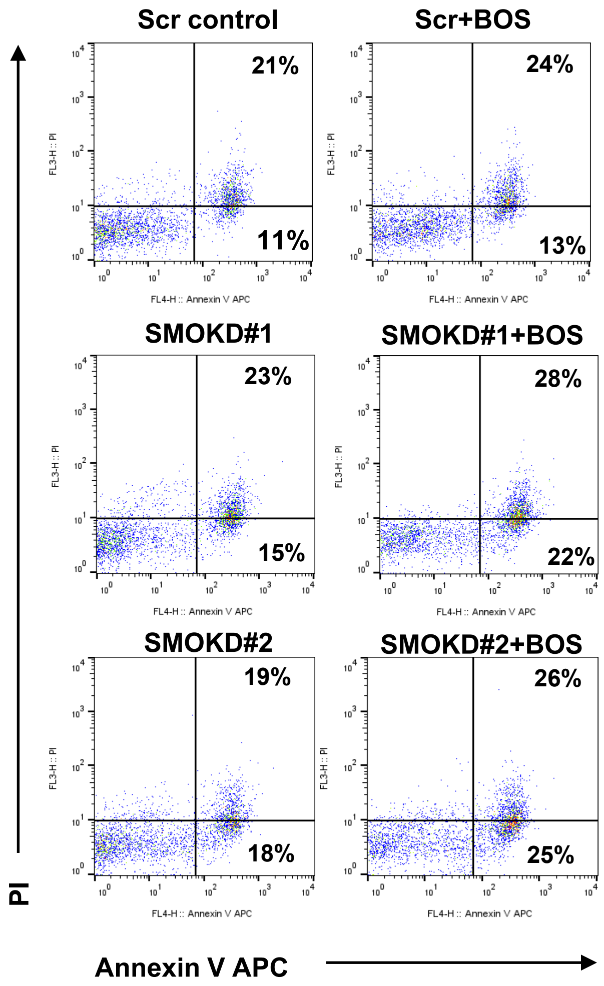

Figure S5. Knockdown of SMO with BOS treatment increased apoptosis in CD34$^+$ IM-nonresponder cells. Representative FACS profiles in CD34$^+$ cells knocked down with two different constructs, with or without treatment of BOS for 72 hours, using PI and Annexin V staining, followed by FACS analysis from an IM-nonresponder patient. scramble control=Scr.
